# Aborted Sudden Cardiac Death in a Young Adult: An Exceptionally Rare Cause

**DOI:** 10.7759/cureus.11013

**Published:** 2020-10-18

**Authors:** Pedro Teixeira, Marisa Silva

**Affiliations:** 1 Cardiology, Centro Hospitalar Vila Nova De Gaia, Vila Nova de Gaia, PRT

**Keywords:** alcapa, sudden cardiac death, anomalous coronary arteries

## Abstract

Out-of-hospital cardiac arrest has an underlying cardiac cause in the vast majority of adult patients, most frequently related to an acute manifestation of atherosclerotic coronary artery disease. Nonetheless, it represents a relatively uncommon event in young adults and athletes, and a thorough investigation of less frequent causes is warranted in this subset. Anomalous origin of a coronary artery is an important, under-recognized, cause of sudden cardiac death in young patients and athletes. Anomalous left coronary artery from pulmonary artery (ALCAPA) is an exceptionally rare variant of these congenital coronary artery origin and/or trajectory anomalies. This case reports the association of ALCAPA with sudden cardiac death in a young patient and highlights some important diagnostic and therapeutic challenges.

## Introduction

Out-of-hospital cardiac arrest (OHCA) is reported to have a cardiac cause in up 80% of patients [1]. Among those, the vast majority have atherosclerotic coronary artery disease, and in this context OHCA is usually triggered by coronary plaque rupture or fissuring (in up to 95% of the cases) leading to acute occlusion. Other less frequent causes include structural heart disease, electrolyte disorders, inherited channelopathies, and trauma. OHCA in young adults and athletes is relatively uncommon and warrants a thorough investigation of even less frequent causes. Anomalous origin of a coronary artery (AOCA) has been proposed as the second most frequent cause of sudden cardiac death (SCD) in young athletes, following hypertrophic cardiomyopathy [2], and was present in 0.6% of 5,100 consecutive cases of SCD [3]. Among AOCA variants, anomalous left coronary artery from pulmonary artery (ALCAPA) is an exceptionally rare entity, with only a minority of patients surviving into adulthood. SCD attributable to myocardial ischemia in ALCAPA patients has been rarely reported [4].

## Case presentation

A 28-year-old sporty (martial arts practitioner) woman, without relevant medical history, activated the national emergency system because of two episodes of pre-syncope at home. Shortly after arrival of the medical team, she collapsed, and the cardiac monitorization showed ventricular fibrillation (VF) from which she was promptly defibrillated, and return of spontaneous circulation ensued. The electrocardiogram (ECG) after defibrillation showed sinus tachycardia with voltage criteria for left ventricular (LV) hypertrophy.

At the emergency room, blood pressure was 105/72 mmHg and a grade II/VI holosystolic murmur was heard at the left sternal border on cardiac auscultation. High-sensitivity cardiac troponin showed a slight elevation (0.045 ng/mL).

Transthoracic echocardiographic evaluation revealed LV enlargement (112 mL/m2), mild LV hypertrophy (maximal wall thickness 12 mm), hypokinesia of the basal and mid segments of the anterior and anteroseptal walls, as well as of all distal segments, and moderately depressed LV function. 

**Figure 1:**
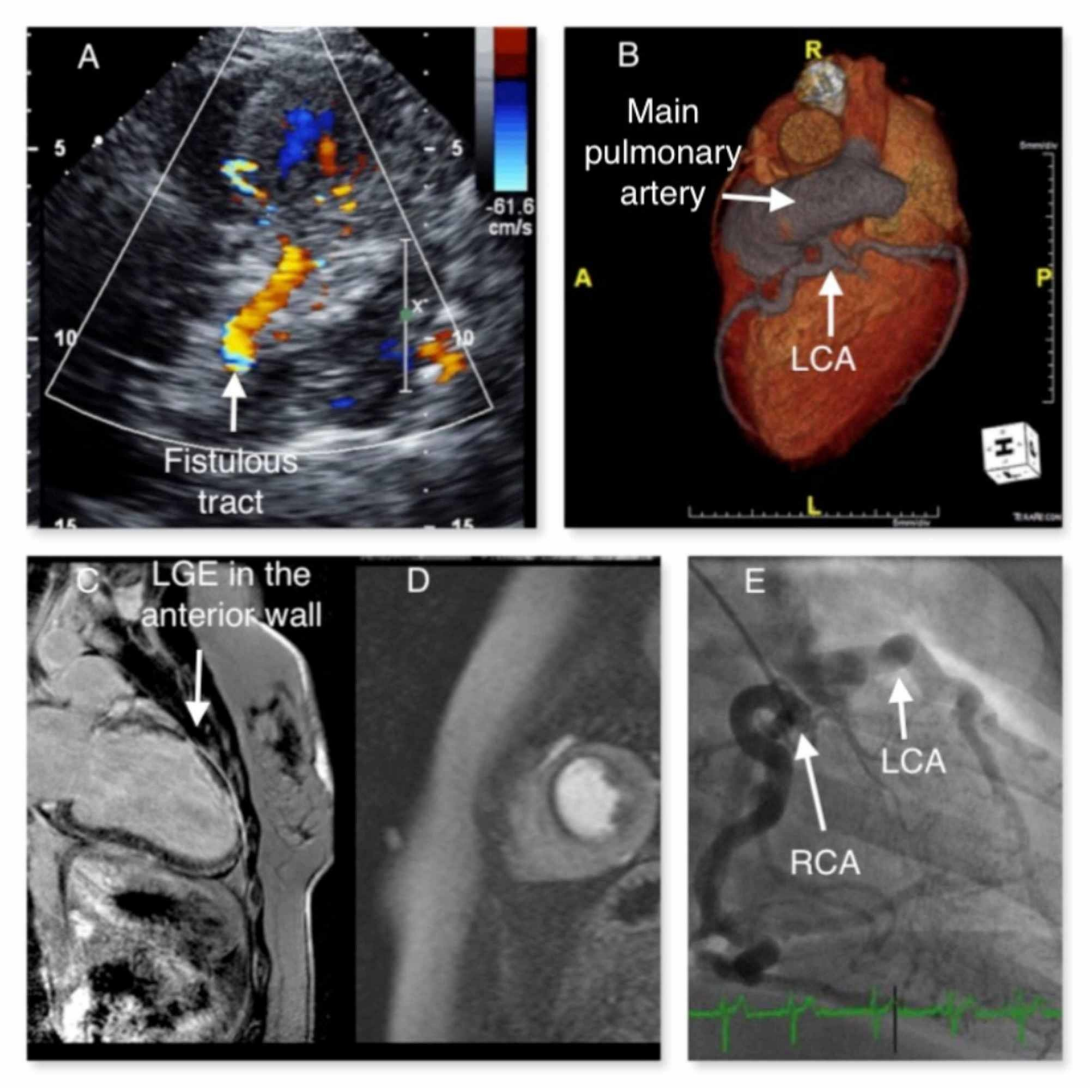
Multimodality imaging approach is crucial to plan the treatment strategy. (A) Fistulous tracts along interventricular septum; (B) Volume-rendered (VR) 3D images showing the anomalous origin of a dilated left coronary artery (LCA) from the main pulmonary artery (MPA); (C) Segmented inversion-recovery gradient echo sequence images after injection of gadolinium showing subendocardial late gadolinium enhancement (LGE) in the anterior wall; (D) Intravenous adenosine infusion reveals peri-infarct ischemia in the middle portions of anterior and septal walls, and remote ischemia in the lateral wall; (E) elective angiogram of the right coronary artery (RCA) showing dilated RCA with collaterals filing the LCA.

The patient underwent coronary CT angiography that showed an anomalous origin of the left coronary artery (LCA) from the pulmonary artery (ALCAPA). This was associated with a dilated right coronary artery (RCA) with extensive collateral network (Figure 1B). Cardiac magnetic resonance (CMR) imaging demonstrated subendocardial late gadolinium enhancement in the anterior wall and mid-distal anteroseptum, with transmural extension in the medium segment of anterior wall (Figure 1C). Furthermore, cardiovascular magnetic resonance imaging (CMR) showed extensive peri-infarct ischemia involving anterolateral wall, middle segment of inferoseptal wall and distal segment of the inferior wall (Figure 1D).

An invasive coronary angiography was performed, and retrograde filling of the LCA territory through collaterals arising from an enlarged RCA was apparent (Figure 1E).

**Figure 2:**
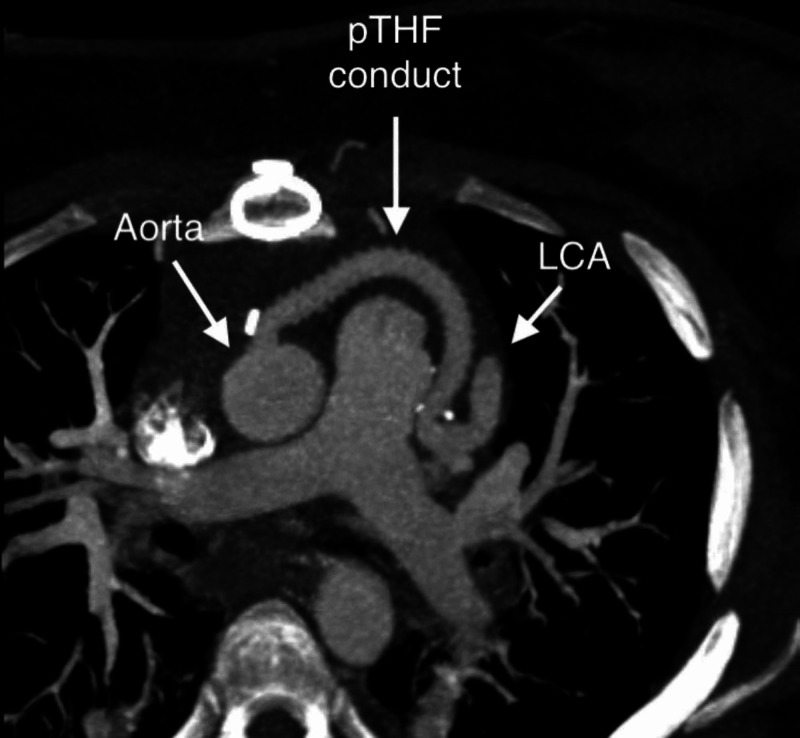
Poly-tetrahydrofuran (pTHF) conduct connecting left coronary artery (LCA) and aorta and bovine pericardial patch closing communication of LCA with main pulmonary artery.

During hospitalization the patient remained hemodynamically stable. She underwent surgical treatment by the creation of a poly-tetrahydrofuran (pTHF) conduct connecting the aorta and left coronary artery (Figure 2). Communication between the main pulmonary artery and left coronary artery was closed with bovine pericardial patch. A transvenous implantable cardioverter defibrillator (ICD) was implanted, for secondary prevention of SCD. She was discharged five days after surgery.

## Discussion

ALCAPA is a rare congenital coronary abnormality, occurring in 1/300000 live births (between 0.24%-0.46% of all congenital cardiac anomalies) and usually presents in the first year of life with congestive heart failure, ischemia, failure to thrive or death [5]. If the undiagnosed patient survives past the first year of life, there is a high likelihood of survival into adulthood. This is possible due to the development of a large network of collateral vessels, sparing the left ventricle from the hypoxic damage [6]. Coronary CT angiography is now the most accurate imaging modality for the detection of congenital coronary artery anomalies [7].

The prognosis is usually determined by the extent of irreversible left ventricular dysfunction and the presence of myocardial scar tissue.

The definitive treatment for ALCAPA is surgical intervention. Surgical correction involves ligation of the anomalous artery combined with coronary artery bypass grafting, an intrapulmonary tunnel repair from the left coronary artery to the aorta (the Takeuchi procedure), or direct reimplantation of the anomalous artery from the pulmonary trunk to the aortic sinus. Direct coronary reimplantation was not possible in this case because of its critical dependence on the distance between the origin of the anomalous artery and the aorta. Nonetheless, it must be noted that it is feasible in the majority of the cases, being the preferred technique [8]. Data on long-term prognosis of these patients is still limited. Concerning myocardial ischemia, heart failure, and aborted SCD, all relevant management strategies with proved prognostic benefit apply [9-11].

## Conclusions

Anomalous origin of a coronary artery is an important, under-recognized, cause of sudden cardiac death in young patients. Among its variants, ALCAPA is an exceptionally rare entity, with only a minority of patients reaching adulthood. This case highlights the diagnostic and therapeutic challenges in this complex clinical field. An integrative multimodality imaging approach is crucial to fully characterize the underlying coronary artery anomalies and plan the management of these most challenging patients. Following ALCAPA diagnosis, surgical management is warranted. Patients' clinical course may be punctuated by different manifestations of myocardial ischemia, heart failure, and aborted SCD, and multidisciplinary patient-centered care in tertiary centers is advised.
